# Prevalence of Pre-Diabetes across Ethnicities: A Review of Impaired Fasting Glucose (IFG) and Impaired Glucose Tolerance (IGT) for Classification of Dysglycaemia

**DOI:** 10.3390/nu9111273

**Published:** 2017-11-22

**Authors:** Wilson C. Y. Yip, Ivana R. Sequeira, Lindsay D. Plank, Sally D. Poppitt

**Affiliations:** 1 Human Nutrition Unit, School of Biological Sciences, University of Auckland, Auckland 1010, New Zealand; i.sequeira@auckland.ac.nz; 2 High-Value Nutrition National Science Challenge, Auckland 1010, New Zealand; s.poppitt@auckland.ac.nz; 3 Department of Surgery, University of Auckland, Auckland 1010, New Zealand; l.plank@auckland.ac.nz; 4 Department of Medicine, University of Auckland, Auckland 1010, New Zealand

**Keywords:** obesity, type 2 diabetes, prediabetes, fasting plasma glucose, oral glucose tolerance test, impaired glucose tolerance, impaired fasting glucose, ethnicity

## Abstract

Prediabetes can be defined by the presence of impaired fasting glucose (IFG) and/or impaired glucose tolerance (IGT), or glycated haemoglobin (Hb_A1c_) to identify individuals at increased risk of developing type 2 diabetes (T2D). The World Health Organization (WHO, 1999) and the American Diabetes Association (ADA, 2003) utilise different cut-off values for IFG (WHO: 6.1–6.9 mmol/L; ADA: 5.6–6.9 mmol/L) but the same cut-off values for IGT (7.8–11.0 mmol/L). This review investigates whether there are differences in prevalence of IFG, IGT, and combined IFG&IGT between ethnicities, in particular Asian Chinese and European Caucasians. In total, we identified 19 studies using the WHO_1999_ classification, for which the average proportional prevalence for isolated (i)-IFG, i-IGT, and combined IFG&IGT were 43.9%, 41.0%, and 13.5%, respectively, for Caucasian and 29.2%, 49.4%, and 18.2%, respectively, for Asian. For the 14 studies using ADA_2003_ classification, the average proportional i-IFG, i-IGT, and combined IFG&IGT prevalences were 58.0%, 20.3%, and 19.8%, respectively, for Caucasian; 48.1%, 27.7%, and 20.5%, respectively, for Asian. Whilst not statistically different, there may be clinically relevant differences in the two populations, with our observations for both classifications indicating that prevalence of i-IFG is higher in Caucasian cohorts whilst i-IGT and combined IFG&IGT are both higher in Asian cohorts.

## 1. Introduction

**Figure 1:**
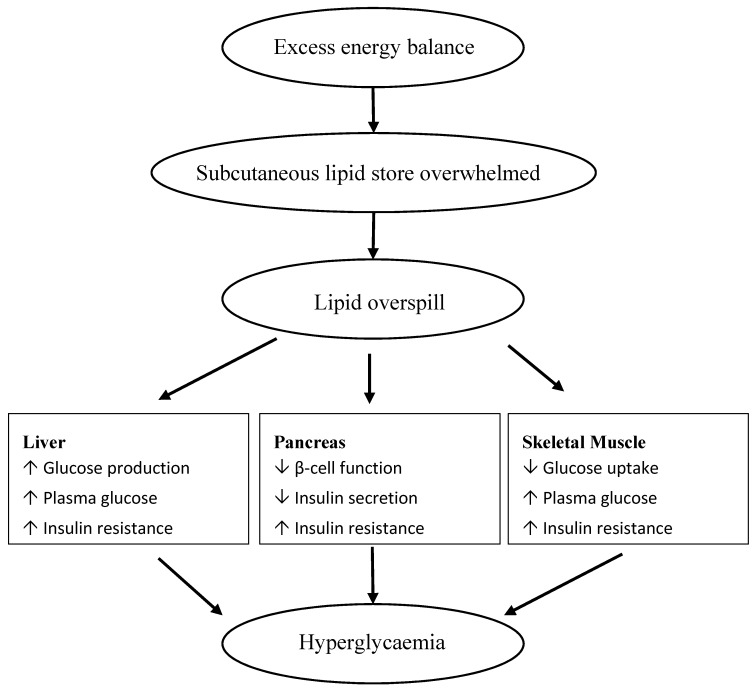
Proposed hypothesis of lipid overspill from subcutaneous adipose into ectopic sites leading to insulin resistance and type 2 diabetes (T2D) [14].

Type 2 diabetes (T2D) is a major health concern worldwide and is increasing in parallel with the obesity epidemic [1]. Prevalence of T2D has increased dramatically with 1 million people reported to have been diagnosed with T2D in 1994, increasing to 382 million by 2013, and with prediction of 592 million by 2035 [2]. T2D is responsible for the deaths of approximately 1.5 million people annually and is a risk factor for cardiovascular disease (CVD), which kills 13 million people worldwide each year, accounting for 25% of all deaths [3], thereby increasing the economic burden within global healthcare systems [4]. Although perceived to be a disease plaguing the more developed nations, such as the countries of Western Europe, North America, and Oceania, T2D prevalence rates have recently been reported to be escalating in developing Asian countries [1], in particular, China [5,6,7,8], and it is estimated that rates will reach 69% by 2030 in comparison to 20% in developed western countries [9]. Arguably, whilst obesity rates are relatively low in terms of population percentage in Asia [10], with 10–25% of the population currently obese across Asian countries [11], there is an alarming number of individuals diagnosed with T2D. For example, in 2013 almost 100 million diagnosed cases of T2D were reported in China [7,12]. The highest prevalence of T2D characteristically is reported within urban settings rather than rural areas [12], given that urbanisation has led to a ‘westernised’ lifestyle with unhealthy food choices and reduced levels of physical activity [5] resulting in a positive energy balance and weight gain [13] with excess lipid accumulated throughout the body (Figure 1).

Given that both genetic and environmental factors contribute to T2D progression, it has been proposed that amongst increasing globalization, Asian ethnicities have been unable to adapt to food and lifestyle related aspects of westernized culture [15]. Hence when matched for the same gender, age, and body weight, those with Asian ethnicity appear to have a greater risk of poor metabolic health than Caucasian counterparts including Europeans, Maori, or Pacific people [16]. This increased risk for T2D has been reported in both northern (Chinese) and southern (Indian) Asians [17]. Recent evidence [18] suggests that it is the differences in body composition, principally fat distribution and partitioning and lean mass content, that are key to understanding the increased susceptibility in Asians to developing adverse metabolic health. For reasons not fully understood, even at a lower body mass index [19] and at lower total adiposity Asians have been shown to have higher abdominal and visceral fat compared to Caucasian counterparts [20]. This difference in fat partitioning has been attributed to increased insulin resistance with concomitant decreased insulin sensitivity in these individuals [21,22]. Furthermore, ectopic fat within visceral abdominal organs such as pancreas and liver as well as muscle [23,24] has also been implicated in the pathophysiology of T2D (Figure 1). Fat infiltration into these organs may alter normal physiological control. Briefly, fatty liver results in hepatic insulin resistance, suppression of gluconeogenesis, and increased circulating blood glucose levels [25]. To maintain glucose homeostasis greater secretion of insulin is required from the pancreatic β-cells, and hence hyperinsulinemia develops. Prolonged hyperinsulinemia and/or fatty pancreas may in turn lead to the dysfunction of pancreatic β-cells, resulting in impaired insulin secretion [26]. Decreased insulin secretion and concomitant increased blood glucose levels consequently also lead to the reduced uptake of glucose by skeletal muscle, thereby enhancing muscle insulin resistance [27].

It has further been shown that Asians have a greater propensity to accumulate ectopic fat, a mechanism considered to be a downstream consequence of ‘impaired’ subcutaneous fat storage [28]. The increased risk of T2D per unit increase in body mass index (BMI) has been suggested to be due to the reduced capacity in Asians to store fat in primary superficial subcutaneous adipose tissue compartments, resulting in the ‘overspill’ into secondary deep subcutaneous and visceral fat compartments [29]. Alternatively, it has been suggested that lipid ‘overspill’ is a consequence of limited fat cell expandability [30], i.e., large adipose cells reach a maximum lipid capacity and are not able to further expand, however, this remains unproven [31]. Notably, the response to nutrition and food may also be different between Asians and Caucasians. For example, it has been shown that the postprandial glycaemic responses after the ingestion of glucose and five varieties of rice in an Asian Chinese population was far greater than a European Caucasian group, matched for gender and age [32]. Similar results were also observed following the consumption of white bread, with the authors [21] concluding that the Asian cohort had lower insulin sensitivity than Caucasians. However, there is paucity of comparative dietary or nutritional data between Asians and Caucasians, especially those that are focused on migrant and native populations, which indeed would have a substantial impact on the outcomes [16].

## 2. Identifying Individuals with Prediabetes

Understanding prediabetes may be crucial to reducing the global T2D epidemic and is defined either by the presence of (i) isolated impaired fasting glucose (i-IFG); or (ii) isolated impaired glucose tolerance (i-IGT); or (iii) both IFG and IGT. IFG, determined from a fasting plasma glucose, occurs as a result of poor glucose regulation, resulting in raised blood glucose even after an overnight fast, while IGT is due to an individual being unable to respond to glucose consumed as part of a meal, resulting in increased postprandial blood glucose [33]. While both IFG and IGT contribute to insulin resistance, the former is the result of hepatic insulin resistance [34] while the latter is primarily the result of insulin resistance in skeletal muscle [35]. Notably, pancreatic β-cell dysfunction is common to both IFG and IGT.

More recently, prediabetes has also been identified by mildly elevated glycated haemoglobin A_1c_ (HbA_1c_) [36,37]. However, given the decreased sensitivity and/or specificity to detect low/intermediary levels of dysglycaemia that characterise prediabetes [38], it has been suggested that HbA_1c_ be used in conjunction with a fasting plasma glucose or oral glucose tolerance test (OGTT) to improve its diagnostic accuracy [39]. Thus, IFG and IGT remain the current recommendations for the identification/diagnosis of prediabetes. Notably, there is as of yet no single definition for prediabetes that is universally accepted by either the research or public health community. This has led to major differences in numbers of individuals classified as high risk prediabetic in different countries, and has slowed progress in determining who should be ‘fast-tracked’ into prevention programs [40]. Identification has primarily been based on guidelines formulated by the World Health Organization (WHO) in 1999 and the American Diabetes Association (ADA) in 2003, both of which provide the same cut point for IGT but different cut points for IFG values [41]. Both WHO_1999_ and ADA_2003_ recommend cut points for IGT as 7.8–11.0 mmol/L measured at the 2 h time point of an OGTT (Table 1). In terms of IFG, WHO_1999_ originally set the cut point for IFG as 6.1–6.9 mmol/L in 1999 [42], which was later revised and lowered to 5.6–6.9 mmol/L by ADA_2003_ in 2003 [43].

The diagnostic criteria for prediabetes based on IFG and IGT cut points have not been changed in the recent guidelines published by ADA in 2016 [33]. ADA_2003_ revised and lowered the IFG cut point in order to improve concordance of diagnosis between both IFG and IGT, with this proposed diagnostic cut off derived from receiver operating characteristic curves of different levels of fasting plasma glucose. This lowered cut point by ADA_2003_ reported an increased global prevalence of individuals defined with prediabetes [44,45,46,47], and whilst it increased the sensitivity of the test it was thought to greatly reduce its specificity [44]. However, the lower IFG cut-off has been challenged by WHO, with progression rate to T2D in those with hepatic insulin resistance (IFG) less than those with skeletal muscle insulin resistance (IGT) [48]. The Diabetes Epidemiology: Collaborative analysis of Diagnostic Criteria in Europe (DECODE) group, under the European Diabetes Epidemiology Group, was subsequently established to evaluate the consequences of the revised diagnostic criteria [49] and concluded that IFG and IGT differ with respect to prediction of all-cause mortality, and cardiovascular morbidity and mortality [50]. Over the years research studies have used either of the prediabetic cut-offs when identifying prediabetic individuals. In recent years, nearly all clinical studies included the ADA_2003_ IFG values in identifying prediabetes [51,52,53,54,55,56,57,58,59,60]. Some studies allow both IFG and/or HbA_1c_ when screening for prediabetic participants [51,56,57,59], while some studies have required both IFG and/or IGT for recruitment [52,54,55,58,60]. It is possible that different ethnicities may predominate as either IFG or IGT, and as such may suggest different sites of impairment of insulin resistance at liver or skeletal muscle between ethnic groups [48]. The aim of this review was to investigate whether there are differences in prevalence of IFG, IGT, and combined IFG&IGT between ethnicities, in particular Asian Chinese and European Caucasians.

## 3. Methods

**Figure 2:**
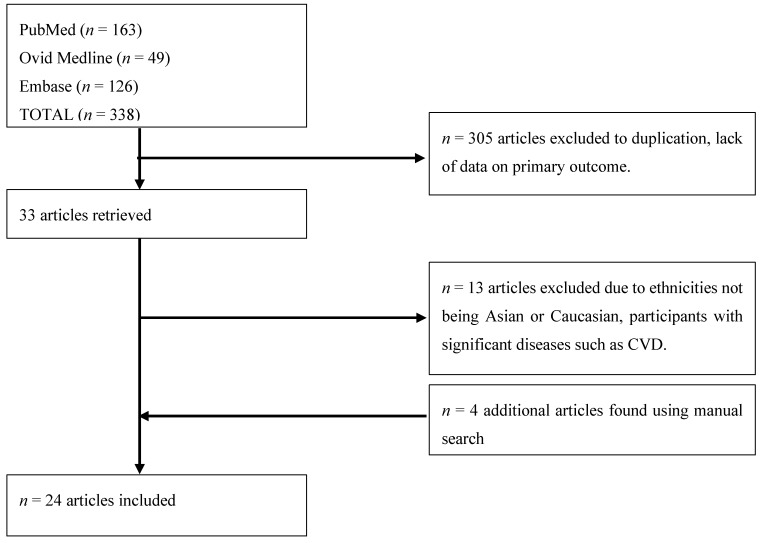
Flow-chart of study selection process. CVD: cardiovascular disease.

The search for relevant studies was undertaken by W.C.Y.Y using electronic databases PubMed, Medline, and Embase up to 31 March 2017. The search terms included the words ‘prevalence’, ‘prediabetes’, ‘impaired fasting glucose’ or ‘IFG’, and ‘impaired glucose tolerance’ or ‘IGT’. A total of 338 articles were found (Figure 2). Only studies that published results of the prediabetic cut-off for both FPG and 2 h glucose at OGTT classified by IFG, IGT, and/or both were included. Both units, mmol/L and mg/dL, were accepted, and studies published as mg/dL were converted to mmol/L. Those with T2D were not included in this analysis. Isolated IFG (i-IFG), isolated IGT (i-IGT), and combined IFG&IGT values were required to be available within the published articles to be included. A total of 305 articles were excluded due to duplicates or non-relevant articles lacking required prevalence data (Figure 2).

The remaining 33 articles were reviewed. Only studies with Caucasian or Asian ethnicity were included, with Caucasians selected from studies published in North American or European cohorts, or studies that specified Caucasian or European ethnic groups. Asians were selected from studies conducted in East Asian, South-East Asian, and South Asian cohorts, or studies in western countries that specified Asian ethnic groups.

Studies were excluded if the participants involved were adolescents or children, or focused on a specific group with significant disease such as CVD. Thus, a further 13 articles were excluded. A manual hand search method to search on similar articles was used on relevant articles identified and reference lists from relevant studies were also reviewed. The manual hand search added four articles, hence a total of 24 articles were included (Figure 2).

Of the 24 articles included, six reported on Chinese cohorts [61,62,63,64,65,66], three Indian cohorts [67,68,69], two Malaysian [70,71], one Thai [72], and one Korean cohort [73]. All were classified as Asian ethnicity. One additional article assessed Asians across China, Japan, India, Indonesia, Singapore, Taiwan and included Asians in Hawaii and Los Angeles, United States [74]. One study reported on a German cohort [75], one French [44], one Italian [47], one Dutch [76], and one Danish cohort [77], in addition to one further study which specified European [78]. All were classified as Caucasian. Again, in two studies conducted in the US, the cohort was classified as Caucasian [79,80]. One study reported on both Asian and European cohorts, with the European data classified as Caucasian [81].

Data were analysed using the statistical software Comprehensive Meta Analysis V3 (Biostat, Inc., Englewood, NJ, USA). Studies from WHO_1999_ were analysed using a random effects model. Studies from ADA_2003_ were also analysed using a random effects model with weighting depending on each study sample size and comparison between the two ethnic groups. Sub-group analysis to address the heterogeneity within and across the ethnic groups was assessed using the *I*^2^ test. High statistical heterogeneity was defined as greater than 70%, medium heterogeneity as 50–70%, and low heterogeneity as 0–50%. Publication bias was assessed by the Begg-Mazumdar [82] and Egger et al. [83] bias indicators.

Data are presented in tables as total number of individuals with prediabetes (*n*), percentage of individuals identified as prediabetic based on isolated-IFG (i-IFG, %), percentage of individuals identified as prediabetic based on isolated-IGT (i-IGT, %), and percentage of individuals identified as prediabetic based on requirement for both IGT and IFG (%). Forest plots are presented as average proportional prevalence taking into account weighting of each study, with the upper and lower error bars calculated with 95% confidence interval (CI) and heterogeneity measured with Tau^2^, Chi^2^, degrees of freedom (*df*) and total *I*^2^ level.

## 4. Results

Twenty-four studies were identified (Table 2 and Table 3) and included in the analysis, of which one study reported both Caucasian and Asian cohorts [81], and eight studies included both the WHO_1999_ and ADA_2003_ prediabetes classifications [44,47,61,65,68,77,79,80]. Most of the studies included were population-based, with a few that were community-based [66,72,73,78], whilst three others were conducted in participants recruited at their work place [47,62,81]. Caucasians include countries of North America and Europe; Asian countries include China, India, Malaysia, Korea, and Thailand. In total, there were 19 studies using the WHO_1999_ classification, of which eight studies focused on Caucasians [44,47,76,77,79,80,81,84] and 11 on Asians [61,62,64,65,66,67,68,69,71,74,81] (Table 2). Fourteen studies utilised the ADA_2003_ classification, with seven Caucasian [44,47,75,77,78,79,80] and seven Asian studies [61,63,65,68,70,72,73] (Table 3). There were a total of 32,204 individuals classified with prediabetes from 19 studies (Table 2), of which 10,999 were Caucasian (eight studies) and 21,205 were Asian (11 studies), based on the WHO_1999_ classification; and 27,112 individuals classified with prediabetes from 14 studies (Table 3), of which 11,744 were Caucasian (seven studies) and 15,765 were Asian (seven studies) based on the ADA_2003_ classification.

All studies included prediabetes of both female and male participants. Participants were more than 20 years old in all studies, with the mean age ranging from 38 to 60 years, and 17 of the 22 studies had a mean age greater than 49 years. The mean BMI for studies involving Caucasians ranged from 26 to 31 kg/m^2^ while those of Asians were 23 to 27 kg/m^2^. However, 11 of the studies did not specify the mean age or BMI (Table 2 and Table 3).

Publication bias was assessed via the Begg-Mazumdar and Egger bias indicators. There was no evidence of publication bias in articles reporting on the prevalence of WHO_1999_ i-IFG in Asians, as the *P* value of each indicator was 0.94 and 0.97, respectively; while *P* values for WHO_1999_ i-IGT in Asians were 0.70 and 0.44, respectively; and *P* values for WHO_1999_ combined IFG&IGT in Asians were 0.59 and 0.99, respectively. Similarly, for articles reporting on the prevalence of WHO_1999_ i-IFG in Caucasians the *P* value of each indicator was 1.00 and 0.65, respectively; while *P* values for WHO_1999_ i-IGT in Caucasians were 0.46 and 0.63, respectively; and *P* values for WHO_1999_ combined IFG&IGT in Caucasians were 0.22 and 0.23, respectively. Again, there was no evidence of publication bias in articles reporting on the prevalence of ADA_2003_ i-IFG in Asians, as the *P* value of each indicator was 0.45 and 0.59, respectively; while *P* values for ADA_2003_ i-IGT in Asians were 0.29 and 0.76, respectively; and *P* values for ADA_2003_ combined IFG&IGT in Asians were 0.88 and 0.56, respectively. Similarly, articles reporting on the prevalence of ADA_2003_ i-IFG in Caucasians listed the *P* values of each indicator as 0.65 and 0.20, respectively; while *P* values for ADA_2003_ i-IGT in Caucasians were 0.45 and 0.89, respectively; and *P* values for ADA_2003_ combined IFG&IGT in Caucasians were 0.45 and 0.25, respectively.

**Figure 3:**
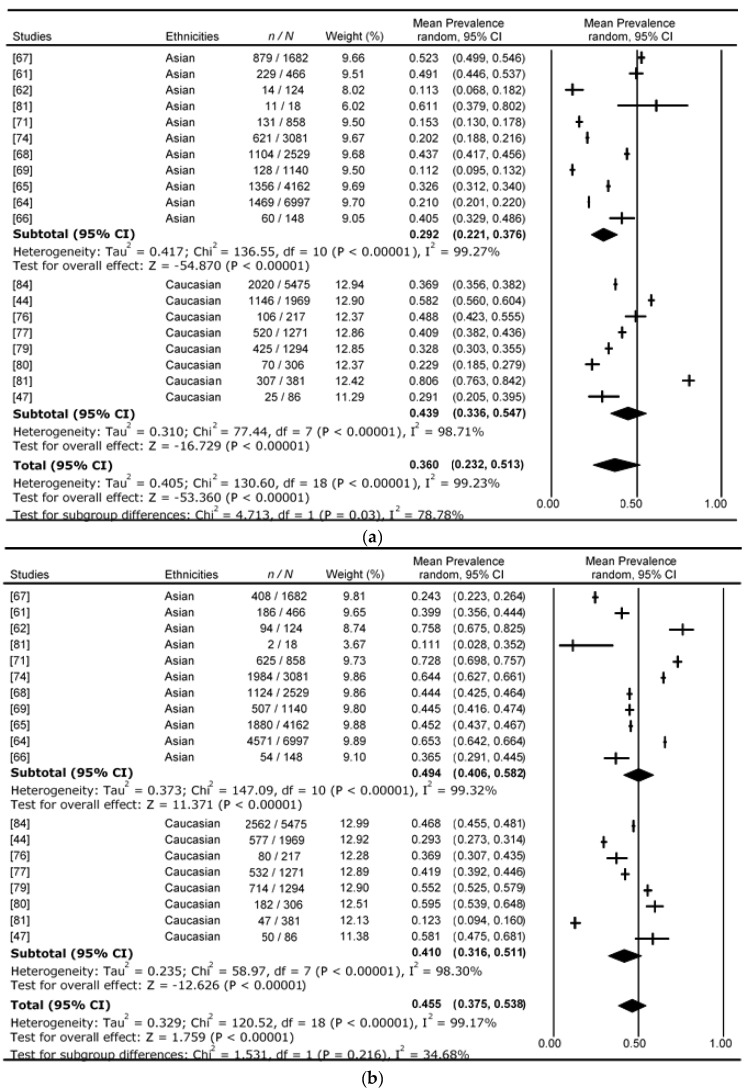

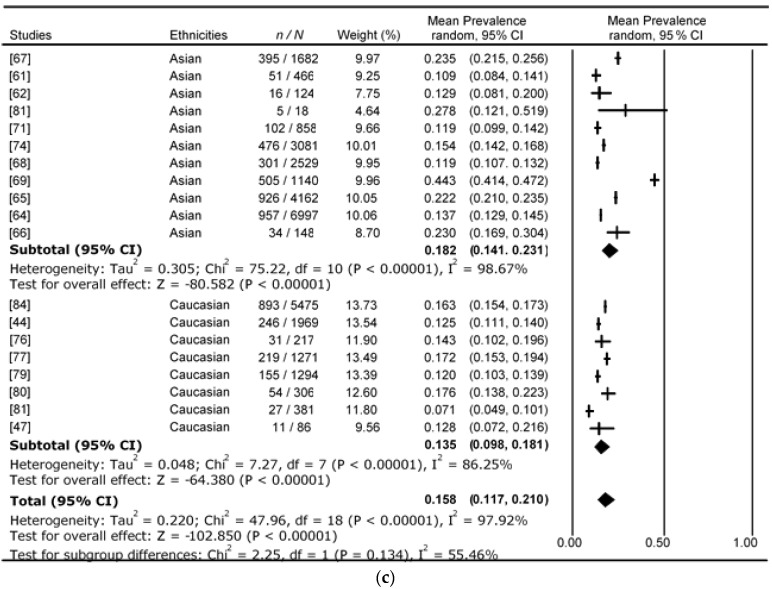
Forest plots for prevalence of prediabetes using WHO_1999_ classification. (**a**) Prevalence of WHO_1999_ i-IFG prediabetic population; (**b**) Prevalence of WHO_1999_ i-IGT prediabetic population; (**c**) Prevalence of WHO_1999_ IFG&IGT prediabetic population.

**Figure 4:**
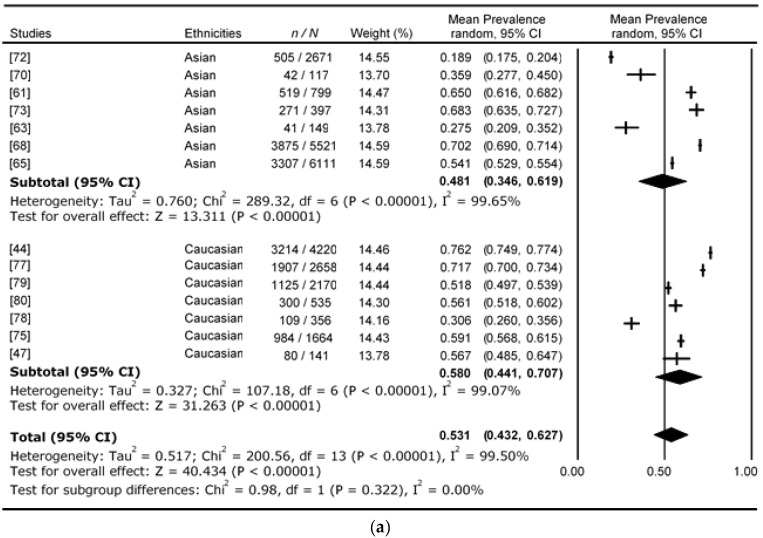

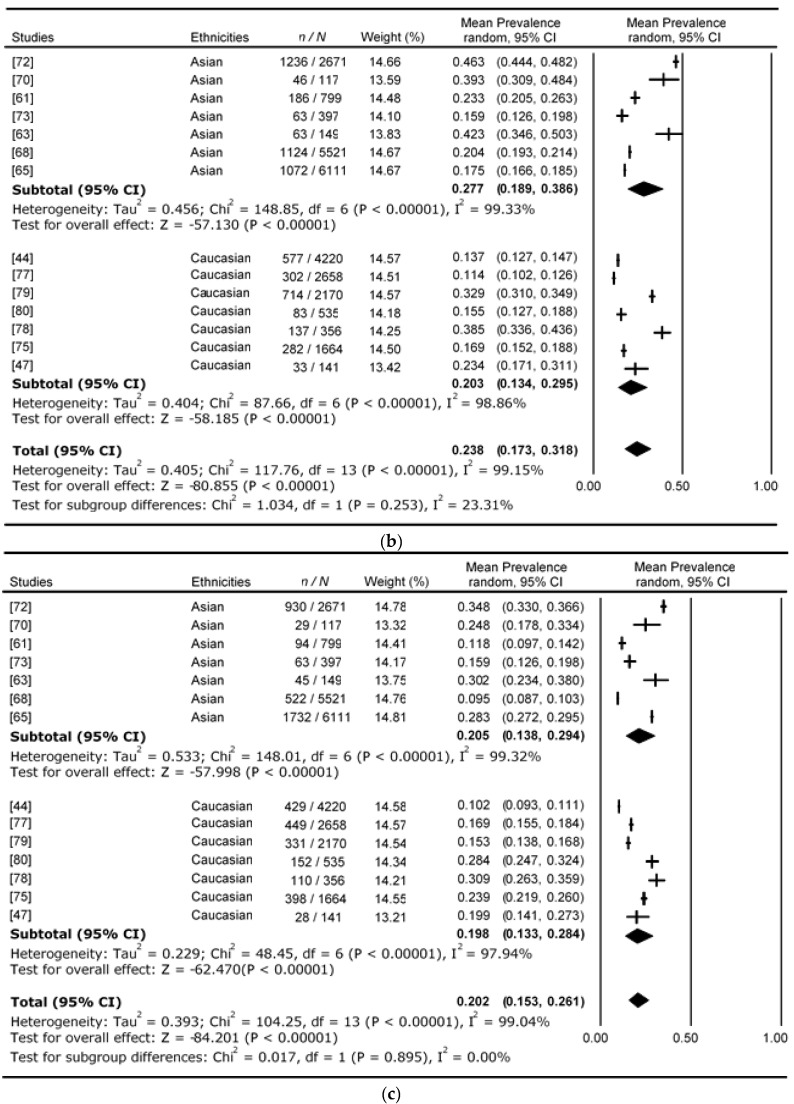
Forest plots for prevalence of prediabetes using ADA_2003_ classification. (**a**) Prevalence of ADA_2003_ i-IFG prediabetic population; (**b**) Prevalence of ADA_2003_ i-IGT prediabetic population; (**c**) Prevalence of ADA_2003_ IFG&IGT prediabetic population.

Figure 3 presents the 19 studies using the WHO_1999_ prediabetes classification. The average proportional prevalence for i-IFG, i-IGT, and combined IFG&IGT were 36.0%, 45.5%, and 15.8%, respectively. IGT is the most common form of dysglycaemia. Figure 4 presents the 14 studies using ADA_2003_ prediabetes classification. The average proportional prevalence for i-IFG, i-IGT, and combined IFG&IGT were 53.1%, 23.8%, and 20.2%, respectively. The prevalence of prediabetic individuals with i-IFG increased from 36.0% to 53.1% with the change of IFG cut-off lowered from 6.1–6.9 mmol/L to 5.6–6.9 mmol/L. This is almost a 20% increase from WHO_1999_ to ADA_2003_, and IFG is the most common form of dysglycaemia according to the ADA_2003_ classification. As a result, the prediabetes prevalence of those with i-IGT is lowered by more than 20% from 45.5% to 23.8% from WHO_1999_ to ADA_2003_ classification.

When looking at the heterogeneity of the WHO_1999_ prediabetes classification among ethnicities, both the Asian and Caucasian studies have high heterogeneity. The total *I*^2^ for i-IFG, i-IGT, and combined IFG&IGT in Asians was 99.3%, 99.3%, and 98.7%, respectively, and in Caucasians 98.7%, 98.3%, and 86.3%, respectively (Figure 3 and Figure 4). The high heterogeneity identifies large variation across studies within each ethnic group. The high heterogeneity is also seen in the ADA_2003_ classification, with total *I*^2^ for i-IFG, i-IGT, and combined IFG&IGT in Asians of 99.7%, 99.3%, and 99.3%, respectively, and in Caucasians 99.1%, 98.9%, and 97.9%, respectively (Figure 3 and Figure 4). In addition to the small number of studies, high heterogeneity may contribute to the asymmetry in funnel plots observed during our analysis.

The prevalences of prediabetes between ethnicities based on the WHO_1999_ classification for i-IFG, i-IGT, and combined IFG&IGT are also shown in Figure 3**,** with data from 19 studies. The average proportional i-IFG for the WHO_1999_ prediabetes classification was 43.9% (95% CI: 33.6–54.7) and 29.2% (95% CI: 22.1–37.6) for Caucasians and Asians, respectively; this is statistically significant (*P* = 0.03 and heterogeneity total *I*^2^ = 78.8%); there is almost 15% greater prevalence of IFG in the Caucasian cohorts. The average proportional i-IGT was 41.0% (95% CI: 31.6–51.1) and 49.4% (95% CI: 40.6–58.2) for Caucasians and Asians, respectively, whilst not statistically significant due to the small number of studies reporting these data (*P* = 0.22 and heterogeneity total *I*^2^ = 34.7%), Asians had 8% higher prevalence. The average proportional combined IFG&IGT was 13.6% (95% CI: 9.8–18.1) and 18.2% (95% CI: 14.1–23.1) for Caucasians and Asians, respectively, although with *P* value = 0.13 and heterogeneity total *I*^2^ = 55.5%, these results were not statistically significant.

The prevalence of prediabetes based on the ADA_2003_ classification for of i-IFG, i-IGT, and combined IFG&IGT are also shown in Figure 4**,** with data from 14 studies. The average proportional i-IFG was 58.0% (95% CI: 44.1–70.7) and 48.1% (95% CI: 34.6–61.9) for Caucasians and Asians, respectively. Whilst not a statistically significant difference due to the small number of studies reporting these data (*P* = 0.32 and heterogeneity total *I*^2^ = 0.0%), again there was 10% greater prevalence of IFG in the Caucasian cohorts. The average proportional i-IGT was 20.3% (95% CI: 13.4–29.5) and 27.7% (95% CI: 18.9–38.6) for Caucasians and Asians, respectively, which was not statistically significant (*P* = 0.25 and heterogeneity total *I*^2^ = 23.3%), although Asians had more than a 7% higher prevalence. The average proportional combined IFG&IGT was similar between ethnicities, with 19.8% (95% CI: 13.3–28.4) and 20.5% (95% CI: 13.8–29.4) for Caucasians and Asians, respectively (*P* = 0.90 and heterogeneity total *I*^2^ = 0.0%).

When comparing between the WHO_1999_ and ADA_2003_ classification, i-IFG_WHO_ is lower than i-IFG_ADA_ for both Caucasians and Asians, while i-IGT_WHO_ is higher than i-IGT_ADA_ for both Caucasians and Asians. The combined IFG&IGT is lower for WHO_1999_ than ADA_2003_ for both Caucasians and Asians. IFG is the most common form of dysglycaemia in both Caucasian and Asian cohorts based on ADA_2003_ classification.

## 5. Discussion

Prediabetes is the term used to classify those at risk of developing T2D, identified as dysglycaemic using either iIFG, iIGT, and/or both. However, there are differences in the classification of IFG between WHO cut-off values of 6.1–6.9 mmol/mol and lower ADA cut-off values of 5.6–6.9 mmol/mol [41]. This change by the ADA increased the prevalence of individuals defined with prediabetes globally [44,45,46,47]. This is consistent with findings in our current review, where the prevalence of i-IFG_WHO_ is at 36.0% and increased to 53.1% for i-IFG_ADA_. One of the reasons for this change was an attempt to balance the number of IFG and IGT individuals from the prediabetic population [85,86]. However, it has raised considerable discussion among experts worldwide noting that WHO did not lower IFG cut points as set by the ADA [87,88]. There are conflicting opinions in the attempt to justify the decrease in IFG cut-off values in determining the risk of developing future T2D [85,89,90].

As shown in our current review, ADA_2003_ IFG is now the most common form of dysglycaemia. However, the lower IFG cut-off has been challenged by WHO, with progression rate to T2D in those with hepatic insulin resistance (IFG) less than those with skeletal muscle insulin resistance (IGT) [48]. It has been proposed that skeletal muscle insulin resistance is the primary defect in T2D [91]. While muscle insulin resistance is clearly involved as a major risk factor to developing T2D, it has been argued that hepatic, not muscle, insulin resistance is central to pathophysiology of hyperglycaemia [92]. Furthermore, β-cell function is decreased with fasting hyperglycaemia and impaired β-cell function worsens as FPG increases [27]. Thus, IFG may be an early indication of an initial risk progression towards IGT and T2D.

Despite the absence of statistical significance for IFG, IGT, or combined IFG&IGT between ethnicities in our current review, other than WHO_1999_ i-IFG data, the differences between the two ethnicities appear to be clinically relevant, with as much as 15% difference between the groups for the various classification cohorts. Absence of statistical significance may be a consequence of the small number of published studies presenting prevalence data, with only eight Caucasian and 11 Asian studies using WHO_1999_, and seven Caucasian and seven Asian studies using the ADA_2003_ classification. Also likely to impact on this is the variability in reported values of i-IFG, i-IGT, and combined IFG&IGT for both theWHO_1999_ and ADA_2003_ cut points, dependent in turn on characteristics of the study population such as BMI, age, and gender. Although mean BMI, a metric based on weight and height, was higher in the Caucasian cohorts than their Asian counterparts, it is recognized to be a poor indictor of total body adiposity and does not capture information related to fat storage in different sites. Cut points for overweight have been revised and lowered for Asian populations [93]. BMI ranged from 26 to 31 kg/m^2^ and 23 to 27 kg/m^2^ for Caucasians and Asians, respectively, and even when matched for BMI there may be differences between ethnicities in the prediabetic population. Evidence supports progression to prediabetes at a lower BMI in Asians, likely due to the differences in body composition and possibly response to nutrient intake [16,32]. Asians may be prone to the Thin on the Outside but Fat on the Inside, ectopic fat ‘TOFI’ phenotype which predisposes to T2D [94]. Age was also highly variable across the studies included in our review, with both young, middle-aged, and elderly cohorts included (summarized in Table 2 and Table 3).

Both WHO_1999_ and ADA_2003_ prediabetes classifications showed higher prevalences for i-IFG among the Caucasian cohort compared to the Asian cohort. This could be explained in part by the western diet commonly characterised as high in fat content and high glycaemic index (GI) carbohydrate load/simple sugars, potentially resulting in increased ectopic lipid storage within liver and subsequent hepatic steatosis [95,96] since IFG occurs due to hepatic insulin resistance [35]. For both i-IGT_WHO_ and i-IGT_ADA_ prediabetes prevalence, the Asian cohort was higher than the Caucasian counterpart. Again, this was not statistically significant but indicates possible ethnic differences as both WHO_1999_ and ADA_2003_ showed the same outcome. IGT is the result of insulin resistance in tissue and muscle, resulting in high circulating glucose due to lack of whole body clearance following a meal. One explanation for differences in IGT may be increased levels of skeletal muscle use, i.e., higher exercise levels [97], since physical activity has been linked to insulin sensitivity and lack of exercise leads to skeletal muscle insulin resistance and IGT [98,99]. However, there was little difference in prevalence of IGT between the two ethnic groups. Asians have evolved into a westernised sedentary lifestyle and the gap between the levels of physical activity in Asians and Caucasians may have narrowed in the past decades [100].

One of the limitations of this review is that it presents only a ‘macro’ view of the two ethnic groups, Caucasians and Asians. In particular, heterogeneity within Asian populations, for example, Asian Indian vs. Asian Chinese, or Chinese living in Europe vs. those still in Asia, may lead to different results due to different lifestyles and cultures [5]. In addition, Asian Indian men have been shown to have lower insulin sensitivity, higher waist circumference, and greater percentage body fat compared to Asian Chinese [101] of similar ages. Those living in urban areas may also be different to those in rural areas within the same country [102]. It is worth noting that the ethnicities reported in this review are based on the assumption that European and American studies present data of Caucasians, but it is possible that not all participants were Caucasians.

## 6. Conclusions

In conclusion, whilst not statistically different there may be clinically relevant differences in the two populations, with prevalence of i-IFG higher in Caucasian cohorts and i-IGT and combined IFG&IGT both higher in Asian cohorts. These outcomes are complicated by the lack of clear definition for the dysglycaemic prediabetic condition, the latter being of utility only if successfully identifying those individuals who do progress to T2D. More research is needed to better understand ethnic differences for the prediabetic state and how this may in turn direct clinical and public health resources to those at greatest risk.

## Figures and Tables

**Table 1:** Diagnostic criteria for prediabetes.

Classification	FPG Range for IFG (mmol/L)	2 h Glucose at OGTT Range for IGT (mmol/L)
WHO_1999_ [42]	6.1–6.9	7.8–11.0
ADA_2003_ [43]	5.6–6.9	7.8–11.0

WHO: World Health Organization; ADA: American Diabetes Association; FPG: Fasting Plasma Glucose; OGTT: Oral Glucose Tolerance Test; IFG: Impaired Fasting Glucose; IGT: Impaired Glucose Tolerance.

**Table 2:** Prevalence of prediabetes using the 1999 World Health Organization (WHO_1999_) classification [43].

Studies	Ethnicity	Mean Age (Years)	Mean BMI (kg/m^2^)	Total Number of Prediabetes (*n*)	i-IFG	i-IGT	IFG & IGT
Harris et al., 1998 [79]	Caucasian	56	27.8	1294	32.88%	55.16%	11.96%
Balkau et al., 2000 [84]	Caucasian	-	-	5475	36.89%	46.79%	16.31%
Metcalf et al., 2000 [81]	Caucasian	-	-	381	80.58%	12.34%	7.09%
de Vegt et al., 2001 [76]	Caucasian	-	-	217	48.85%	36.87%	14.29%
Glumer et al., 2003 [77]	Caucasian	49	28.4	1271	40.91%	41.86%	17.23%
Borch-Johnsen et al., 2004 [44]	Caucasian	49	26.0	1969	58.21%	29.29%	12.50%
Vaccaro et al., 2005 [47]	Caucasian	-	-	86	29.07%	58.14%	12.79%
Karve et al., 2010 [80]	Caucasian	49	30.7	306	22.73%	59.60%	17.68%
Ko et al., 1998 [62]	Asian	38	23.3	124	11.29%	75.81%	12.90%
Metcalf et al., 2000 [81]	Asian	-	-	18	61.11%	11.11%	27.78%
Qiao et al., 2000 [74]	Asian	53	23.8	3081	20.16%	64.39%	15.45%
Ramachandran et al., 2001 [68]	Asian	49	23.1	2529	43.65%	44.44%	11.90%
Dong et al., 2003 [61]	Asian	54	26.4	466	49.22%	39.92%	10.85%
Sadikot et al., 2004 [69]	Asian	-	-	1140	11.23%	44.47%	44.30%
Yang et al., 2004 [65]	Asian	-	-	4162	32.57%	45.18%	22.25%
Yang et al., 2010 [64]	Asian	51	25.3	6997	20.99%	65.33%	13.68%
Anjana et al., 2011 [67]	Asian	-	-	1682	52.26%	24.24%	23.50%
Mustafa et al., 2011 [71]	Asian	50	26.9	858	15.27%	72.84%	11.89%
Zhuang et al., 2015 [66]	Asian	52	23.8	148	40.54%	36.49%	22.97%
TOTAL				32,204			

BMI: Body Mass Index; i-IFG: Isolated Impaired Fasting Glucose (6.1–6.9 mmol/L); i-IGT: Isolated Impaired Glucose Tolerance (7.8–11.0 mmol/L); IFG & IGT: Combined Impaired Fasting Glucose and Impaired Glucose Tolerance (6.1–6.9 mmol/L and 7.8–11.0 mmol/L); “-“ information unavailable.

**Table 3:** Prevalence of prediabetes using the 2003 American Diabetes Association (ADA_2003_) classification [44].

Studies	Ethnicity	Mean Age (Years)	Mean BMI (kg/m^2^)	Total Number of Prediabetes (*n*)	i-IFG	i-IGT	IFG & IGT
Harris et al., 1998 [79]	Caucasian	56	27.8	2170	51.86%	32.90%	15.24%
Glumer et al., 2003 [77]	Caucasian	47	27.0	2658	71.75%	11.36%	16.89%
Borch-Johnsen et al., 2004 [44]	Caucasian	49	26.0	4220	76.17%	13.67%	10.17%
Vaccaro et al., 2005 [47]	Caucasian	-	-	141	56.74%	23.40%	19.86%
Karve et al., 2010 [80]	Caucasian	49	30.7	535	56.07%	15.61%	28.32%
Marini et al., 2012 [78]	Caucasian	52	30.6	356	30.62%	38.48%	30.90%
Tamayo et al., 2014 [75]	Caucasian	55	27.7	1664	59.13%	16.95%	23.92%
Ramachandran et al., 2001 [68]	Asian	40	23.1	5521	70.18%	20.36%	9.45%
Dong et al., 2003 [61]	Asian	54	26.4	799	64.93%	23.30%	11.76%
Yang et al., 2004 [65]	Asian	-	-	6111	54.11%	17.55%	28.34%
Lee et al., 2011 [73]	Asian	-	-	397	68.26%	15.87%	15.87%
Chew et al., 2012 [70]	Asian	39	26.9	117	35.90%	39.32%	24.79%
Liu et al., 2014 [63]	Asian	60	23.7	149	27.52%	42.28%	30.20%
Aekplakorn et al., 2015 [72]	Asian	51	26.2	2671	18.91%	46.27%	34.82%
TOTAL				27,112			

BMI: Body Mass Index; i-IFG: Isolated Impaired Fasting Glucose (5.6–6.9 mmol/L); i-IGT: Isolated Impaired Glucose Tolerance (7.8–11.0 mmol/L); IFG & IGT: Combined Impaired Fasting Glucose and Impaired Glucose Tolerance (5.6–6.9 mmol/L and 7.8–11.0 mmol/L); “-“ information unavailable.
